# The value of reducing arthroscopic partial meniscectomy in the treatment of degenerative meniscus tears: a budget impact analysis

**DOI:** 10.1017/S0266462322003361

**Published:** 2023-01-18

**Authors:** Evelien B. van Well, Stan R.W. Wijn, Gerjon Hannink, Janneke P.C. Grutters, Maroeska M. Rovers

**Affiliations:** 1 Department of Medical Imaging, Radboud University Medical Centre, Nijmegen, The Netherlands; 2 Department of Health Evidence, Radboud University Medical Center, Nijmegen, The Netherlands

**Keywords:** arthroscopic partial meniscectomy, degenerative meniscus tear, budget impact analysis, low-value care, health expenditures

## Abstract

**Aims:**

Numerous studies have shown that arthroscopic partial meniscectomy (APM) is not (cost-) effective in patients with symptoms attributed to a degenerative meniscus tear. We aimed to assess the budget impact of reducing APM in routine clinical practice in this population.

**Materials and methods:**

A patient-level state transition model was developed to simulate patients recently diagnosed with a degenerative meniscus tear. Three strategies were compared: “current guideline” (i.e., postpone surgery to at least 3 months after diagnosis), “APM at any time” (i.e., APM available directly after diagnosis), and “nonsurgical” (i.e., APM no longer performed). Total societal costs over 5 years were calculated to determine the budget impact. Probabilistic and deterministic sensitivity analyses were conducted to address uncertainty.

**Results:**

The average cost per patient over 5 years were EUR 5,077, EUR 4,577, and EUR 4,218, for the “APM at any time,” “current guideline,” and “nonsurgical” strategy, respectively. Removing APM from the treatment mix (i.e., 30,000 patients per year) in the Netherlands, resulted in a reduction in health care expenditures of EUR 54 million (95 percent confidence interval [CI] EUR 38 million–EUR 70 million) compared to the “current guideline strategy” and EUR 129 million (95 percent CI EUR 102 million–EUR 156 million) compared to the “APM at any time” strategy. Sensitivity analyses showed that uncertainty did not alter our conclusions.

**Conclusions:**

Substantial costs can be saved when APM is no longer performed to treat symptoms attributed to degenerative meniscus tears in the Netherlands. It is therefore recommended to further reduce the use of APM to treat degenerative meniscus tears.

## Introduction

Knee pain due to degenerative knee disease occurs in 25 percent of people over 50 years, many of whom develop a degenerative meniscus tear (1). Arthroscopic partial meniscectomy (APM) is often performed to alleviate the symptoms thought to be caused by a meniscus tear, such as knee pain and catching and locking of the knee (1;2). However, it is unclear if these symptoms are caused by the meniscus tear itself or the degenerative knee disease in general (3). There are numerous randomized controlled trials (RCTs), meta-analyses, and cost-effectiveness studies that show that APM is not a (cost-)effective treatment for patients with a degenerative meniscus tear (4–6). The procedure is even associated with an increased risk of osteoarthritis progression (7). Based on this accumulated evidence, clinical practice guidelines have advised against the use of APM as a primary treatment. Instead, APM should only be considered when nonsurgical treatment has not provided symptom alleviation (2;8).

Despite these guidelines, the usage of APM to treat degenerative meniscus tears remains high. Countries such as Denmark and the United Kingdom, either show only a small decrease or even an increase in the number of APMs performed in middle-aged and elderly populations in the last decade (9;10). In the Netherlands, approximately 20,000 APMs are performed yearly in patients over 40 years old (11). Numerous orthopedic surgeons remain convinced there are patients with a degenerative meniscus tear who do benefit from APM, even though evidence for such a subgroup is lacking (12;13). Given that APM is not proven effective in patients with a degenerative meniscus tear, costs could be saved if APM is no longer performed to treat these patients. In healthcare systems with fixed budgets, these costs are better spent on proven (cost-)effective treatments. Previous studies have shown APM is not cost-effective, but none of them investigated how much costs could be saved if APM is no longer performed to treat patients with a degenerative meniscus tear (5;14;15). Therefore, we aimed to assess the budget impact of removing APM from routine clinical practice in the treatment of degenerative meniscus tears in the Netherlands.

## Materials and methods

A patient-level state transition model was created to assess the treatment costs of degenerative meniscus tears. Total societal costs were determined every year for 5 years total. Patients were simulated individually allowing the inclusion of treatment history and age. Patients that entered the model were recently diagnosed with a degenerative meniscus tear without a locked knee and no previous injury to the meniscus. The eligible population was open, meaning that every year 30,000 newly diagnosed patients entered the model, increasing the size of the cohort over 5 years. The estimated population size of 30,000 patients per year was based on the incidence of degenerative meniscus tears in the Netherlands (16). This open population allowed for the calculation of the total budget impact over 5 years, as is standard in a budget impact analysis (17).

Three treatment strategies were modeled to assess the budget impact.

*Current meniscus guidelines strategy (postpone surgery to at least 3 months after diagnosis)*: In the first strategy, current Dutch and international guidelines for the treatment of a degenerative meniscus tear were followed, that is, patients were eligible for APM when other nonsurgical treatments did not resolve complaints (at least 3 months after diagnosis) (2;8). Nonsurgical treatment of a degenerative meniscus tear consists of either physical therapy (PT) or physical therapy combined with corticosteroid injections (PT + CORT).

*APM at any time strategy*: Because of the lack of guideline adherence (11), a second strategy was included in which current guidelines were not followed. This strategy represents the situation before the guidelines or when current guidelines are ignored. In this strategy, patients were allowed to undergo APM at any time directly after diagnosis.

*Nonsurgical strategy*: In the third strategy, APM was not performed at all. Instead, patients could receive nonsurgical treatments after diagnosis.

### Model structure

**Figure 1.**
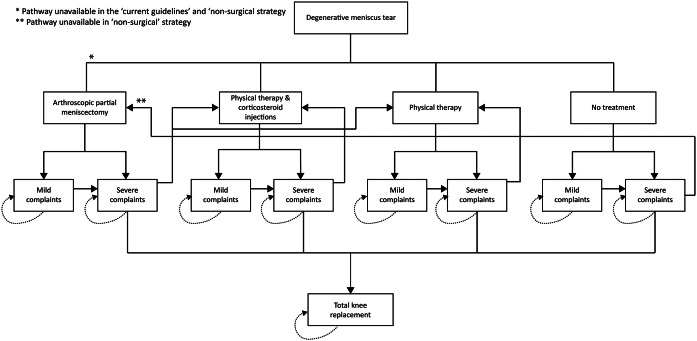
Model structure representing the treatment pathway of all strategies. All patients could transition to the death state (not depicted).

The model consisted of the following health states: mild complaints after every treatment, severe complaints after treatment, total knee replacement (TKR), and death. Figure 1 provides an overview of the model structure. In every strategy, patients could receive PT, PT + CORT, or no treatment. After each treatment, patients could either improve and only experience mild knee complaints or still experience severe complaints if the treatment was not effective. Over time, patients’ complaints could worsen, causing patients in the “mild complaints” health state to transition to the “severe complaints” health state. Patients with severe complaints were able to again undergo one of the above-mentioned treatment options or receive a TKR. Patients that have undergone PT or PT + CORT were able to receive these treatments repeatedly. The assumption was made that patients could only undergo APM and TKR once. We simulated that patients could transition between the health states every 3 months because of treatment durations and waiting time between treatments (8;18). Also, patients could transition to the death state from every health state due to all-cause mortality, based on age-based mortality rates in the Netherlands.

### Probabilities

All probabilities were extracted from literature, except the initial treatment distribution, which was based on expert opinion. The experts consulted were two orthopedic surgeons, with over 15 years of experience. The transitions from treatment to mild or severe complaints were based on an RCT comparing PT and APM (19). PT and PT + CORT treatments were assumed to have similar transition probabilities to mild or severe complaints as corticosteroid injections are mainly used to provide pain relief to allow patients with severe pain complaints to complete PT. According to the experts, patients who choose no treatment can recover, and the probability of this occurrence was based on sham surgery outcomes (20). The transition from mild to severe complaints was based on long-term follow-up of treatment strategies, as patients can experience complaints again or develop new complaints over time (21). The probability to undergo a TKR after standard care and after APM was based on cost-effectiveness studies and studies assessing the increased risk of osteoarthritis progression after APM (5;7). The probability of undergoing another treatment when experiencing severe complaints was based on treatment cross-over in RCTs and expert opinion (19). All probabilities were converted to 3-month probabilities (22). Table 1 shows all probabilities used in the model.

**Table 1.** Three monthly transition probabilities used in the model, including the 95 percent confidence interval using a beta distribution, and source of the parameter

Parameter	Mean	95% CI	Source
*All strategies*			
Mild complaints after APM	.73	.66–.80	(19)
Severe complaints after APM	.27	.20–.34	(19)
Mild complaints after PT + CORT	.47	.40–.55	(19)
Severe complaints after PT + CORT	.53	.45–.60	(19)
Mild complaints after PT	.47	.40–.55	(19)
Severe complaints after PT	.53	.45–.60	(19)
Mild complaints after no treatment	.37	.16–.60	(20)
Severe complaints after no treatment	.63	.42–.84	(20)
Severe complaints after mild complaints	.04	.02–.06	(21)
Severe complaints APM after mild complaints APM	.06	.03–.09	(21)
PT after severe complaints	.11	.08–.13^a^	(19)
PT + CORT after severe complaints	.11	.08–.13^a^	(19)
PT after severe complaints APM	.05	.04–.07^a^	(19)
PT + CORT after Severe complaints APM	.05	.04–.07^a^	(19)
TKR	.005	.004–.006^a^	(5)
TKR with previous APM	.006	.005–.008^a^	(5)
*Current guideline strategy*			
APM as initial treatment	0	–	–
PT as initial treatment	.45	.35–.55^b^	Expert opinion
PT + CORT as initial treatment	.45	.35–.55^b^	Expert opinion
No treatment as initial treatment	.10	.04–.16^b^	Expert opinion
APM after Severe complaints	.11	.09–.14	(19)
*APM at any time strategy*			
APM as initial treatment	.35	.26–.44^b^	Expert opinion
PT as initial treatment	.275	.19–.36^b^	Expert opinion
PT + CORT as initial treatment	.275	.19–.36^b^	Expert opinion
No treatment as initial treatment	.10	0–.16^b^	Expert opinion
APM after severe complaints	.11	.09–.14	(19)
*Nonsurgical strategy*			
APM as initial treatment	0	–	–
PT as initial treatment	.45	.34–.56^b^	Expert opinion
PT + CORT as initial treatment	.45	.34–.56^b^	Expert opinion
No treatment as initial treatment	.10	0–.16^b^	Expert opinion
APM after severe complaints	0	–	–

a No 95 percent confidence interval was available and a standard error of 25 percent of the mean was assumed.

b 95 percent CI using a Dirichlet distribution instead of a beta distribution.

Abbreviations: PT, physical therapy; CORT, corticosteroid injection; APM, arthroscopic partial meniscectomy; TKR, total knee replacement.

### Costs

Costs were assessed from a societal perspective in Euros (EUR). Surgery costs of APM and TKR were based on previous cost-effectiveness studies (5;15). The costs of corticosteroids were obtained from the Dutch pharmacotherapeutic compass, and injection costs were procured from the Dutch guideline of economic evaluations in healthcare (23;24). PT costs were based on average PT costs per session in the Netherlands multiplied by nine sessions, which is the minimal number of PT sessions reimbursed by healthcare insurers, and is in line with the average number of sessions in the PT program of Katz et al. (19;23). Societal costs were included during follow up. These costs were based on a recent cost-effectiveness study, separated for mild and severe complaints (15). Additional medical costs were added to the costs of the severe complaints health state, which were based on the average additional medical costs of osteoarthritis patients (25). Indexation was performed to transform all costs to 2020. All costs are shown in Table 2.

**Table 2.** Cost estimates used in the model, including the 95 percent confidence intervals, the distribution used in probabilistic sensitivity analysis, and source of the parameter

Parameter	Mean cost (95% CI)	Distribution	Source
APM	EUR 2,105	Fixed	(15)
PT	EUR 306 (238–374)	Gamma	(23)
CORT	EUR 68	Fixed	(23, 24)
Mild complaints	EUR 294 (224–365)	Gamma	(15)
Severe complaints	EUR 405 (335–476)	Gamma	(15)
TKR	EUR 16,821	Fixed	(5)

Abbreviations: APM, arthroscopic partial meniscectomy; CORT, corticosteroid injection; PT, physical therapy; TKR, total knee replacement.

### Analyses

The yearly and 5-yearly population costs were compared between the three treatment strategies. To test the robustness of the model, a one-way sensitivity analysis was performed for key variables: the initial treatment distribution, treatment outcomes (mild or severe complaints), and progression from mild to severe complaints over time. These probabilities were varied by a relative 10, 20, and 30 percent in both directions. In addition, the incidence of degenerative meniscus tears in the Netherlands (*n* = 30,000) was varied by 50 percent in both directions due to high uncertainty in this parameter. Parameter uncertainty was taken into account using probabilistic sensitivity analysis (PSA) with 1,000 iterations, that is, the model was executed 1,000 times using randomly sampled input parameters. All probabilities were drawn from a beta distribution or Dirichlet distribution and costs from a gamma distribution. When the confidence interval around a transition probability was unknown, a standard error of 25 percent of the deterministic value was applied.

The model was developed in R (version 4.0.3, the R Foundation for Statistical Computing, Vienna, Austria). Following the AdviSHE checklist, the model was validated by consulting clinical experts, cross-validation, extreme value testing, and trace testing (26).

## Results

The model outcomes, including PSA, show the “APM at any time” strategy was the most expensive strategy, while “nonsurgical” was the least expensive strategy (deterministic results are shown in Supplementary Table 1). The costs per patient were EUR 4,577 for the “current guideline” strategy, EUR 5,077 for the “APM at any time” strategy, and EUR 4,218 for the “nonsurgical” strategy. The “APM at any time” had the highest total 5-year costs of EUR 762 million (95 percent confidence interval [CI]: EUR 677 million–EUR 846 million), followed by the “current guideline” strategy with EUR 687 million (95 percent CI: EUR 603 million–EUR 770 million), and the “nonsurgical” strategy with EUR 633 million (95 percent CI: EUR 554 million–EUR 712 million). Table 3 and Supplementary Figure 1 show the yearly and total costs of all strategies along with the uncertainty, with yearly costs increasing over time as every year a new group of patients is added to the modeled population.

**Table 3.** Yearly and total treatment cost in million Euros per strategy including the 95 percent confidence interval

Costs (95% CI)	Current guideline (EUR)	APM at any time (EUR)	Nonsurgical (EUR)
Year 1	47.67 (43.05–52.29)	63.61 (57.29–69.93)	42.89 (38.44–47.35)
Year 2	95.36 (84.85–105.87)	110.67 (99.49–121.86)	86.78 (76.75–96.82)
Year 3	139.25 (122.56–155.95)	153.29 (136.40–170.17)	128.15 (112.25–144.04)
Year 4	181.99 (159.06–204.91)	196.59 (173.71–219.47)	167.55 (145.93–189.16)
Year 5	222.33 (193.19–251.47)	237.42 (208.78–266.05)	207.38 (179.83–234.92)
Total costs	**686.60 (603.32–769.88)**	**761.57 (677.11–846.04)**	**632.75 (553.60–711.90)**

Abbreviation: APM, arthroscopic partial meniscectomy.

The budget impact of changing from the “APM at any time” strategy to the “current guideline” strategy was a cost reduction of EUR 75 million (95 percent CI: EUR 50 million–EUR 99 million) over 5 years. The budget impact of changing from the “current guideline” strategy to the “nonsurgical” strategy was a cost reduction of EUR 54 million (95 percent CI: EUR 38 million–EUR 70 million) over 5 years. Changing from the “APM at any time” strategy to “nonsurgical” strategy resulted in a cost reduction of EUR 129 million (95 percent CI: EUR 102 million–EUR 156 million) over 5 years. Supplementary Figure 2 shows the budget impact of the “current guideline” strategy and “nonsurgical” strategy compared to the “APM at any time” strategy.

### One-way sensitivity analysis

**Figure 2.**
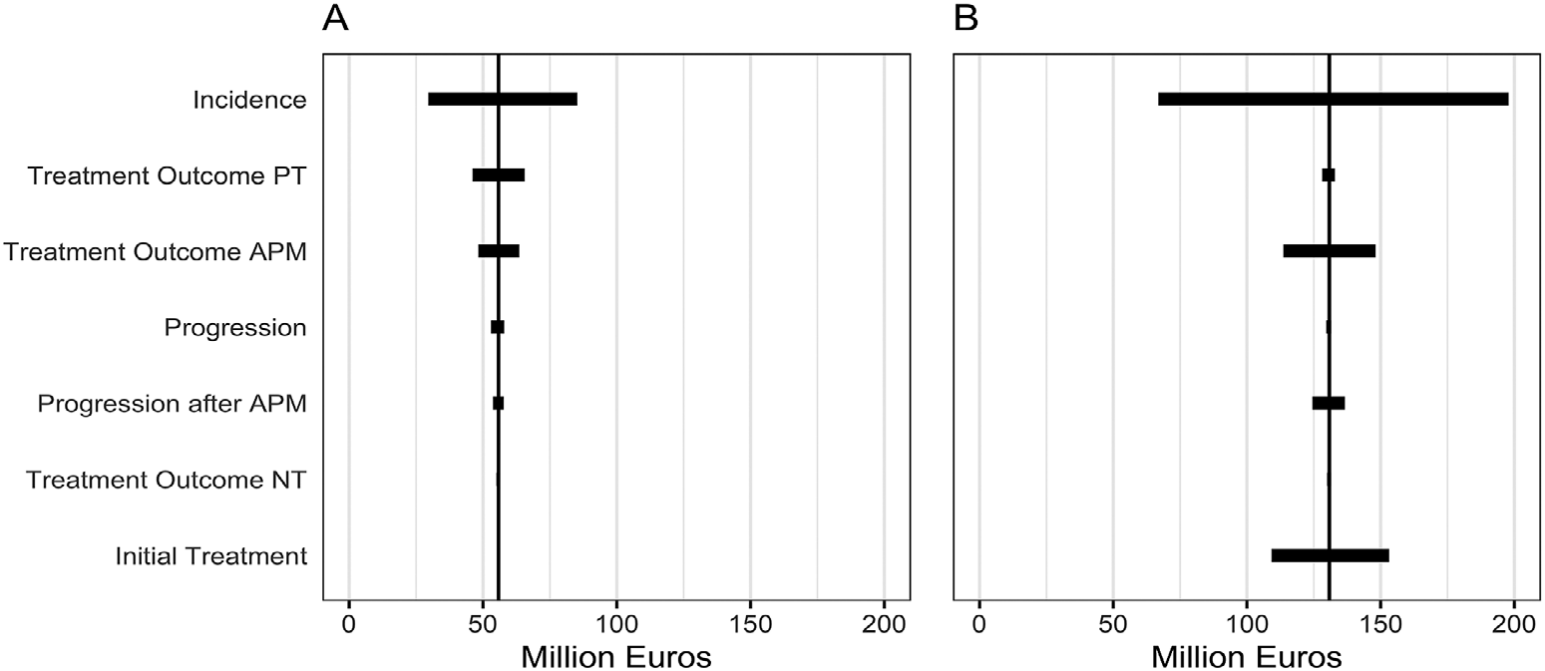
One-way sensitivity analysis of key variables (incidence, treatment outcomes, progression, and initial treatment choice) on the budget impact of (A) the “nonsurgical” strategy compared to the “current guideline” strategy, and (B) the “nonsurgical” strategy compared to the “APM at any time” strategy. Showing the range of the budget impact per parameter when input parameters were varied by 30 percent. Abbreviations: APM, arthroscopic partial meniscectomy; PT, physical therapy; NT, no treatment.

The one-way sensitivity analysis showed that the results of the “APM at any time” strategy were mainly influenced by changes in the initial treatment choice (i.e., the percentage of patients that received APM), PT treatment outcome, and APM treatment outcome (i.e., the percentage patients with mild and severe complains after treatment). The “current guideline” and “nonsurgical” strategies were mainly influenced by changes in the PT treatment outcome (Figure 2). Although these variables influenced the overall costs of the strategies, they only accounted for a maximum of 12 percent variation in the outcome. The incidence of degenerative meniscus tears (number of new patients entering the model each year) had the largest influence on the total cost.

### Interactive web application

A web application was created in which users can adjust the model with alternative input parameters allowing the model to be applied to multiple settings, including other countries. The application was built using the shiny (version 1.7.1) and shinydashboard (version 0.7.2) R-package. The web application is available at: https://stanwijn.shinyapps.io/APM-BIA/.

## Discussion

APMs are still widely performed as a treatment for symptoms attributed to degenerative meniscus tears in the Netherlands. Therefore, we conducted a budget impact analysis to investigate the impact of reducing APM in routine clinical practice in the treatment of degenerative meniscus tears. The analysis showed that changing from “current guideline” strategy to a strategy where APM was removed for 30,000 patients per year in the Netherlands, resulted in a reduction in healthcare expenditures of EUR 54 million (95 percent CI: EUR 38 million–EUR 70 million), while changing from the “APM at any time” strategy to a strategy without APM resulted in a reduction in health care expenditures of EUR 129 million (95 percent CI: EUR 102 million–EUR 156 million). These results were similar in all sensitivity analyses.

Multiple studies show that APM is not cost-effective in this population, this study emphasizes reducing APM as a treatment for degenerative meniscus tears results in costs savings (5;15). Yet, our results are difficult to compare to other literature because similar budget-impact studies are lacking. Previously, two budget-impact analyses were described in protocols: one alongside the ESCAPE study (APM in patients with a degenerative meniscus tear), and one in the STARR trial (aimed to assess APM in patients with a traumatic meniscus tear). However, both are currently not published in the literature (27;28).

### Strengths and limitations

To the best of our knowledge, we are the first to assess the budget impact of reducing APM in the treatment mix of patients with a degenerative meniscus tear. Our model simulated the entire treatment pathway of a degenerative meniscus tear, allowing for multiple treatments and taking into account osteoarthritis progression. Additionally, the web application can be used to calculate the budget impact of reducing APM in other countries (with comparable treatment options) by allowing users to adjust the input parameters of the model. In addition, this allows for adjustments to be made over time, increasing the longevity and relevance of the model.

This study also has some limitations to consider. First, limited data were available on the incidence of degenerative meniscus tears in the Netherlands and the initial treatment choice of patients after diagnosis. These parameters were varied in the one-way sensitivity analysis and PSA, but did not alter our conclusions. Second, some parameters lacked a parameter distribution (uncertainty). For these parameters, we used a standard error of 25 percent to ensure uncertainty was still considered. Third, our model was developed to estimate the budget impact for reducing APM in The Netherlands. This limits the generalizability of the budget impact to other countries. However, the web application can be used to adapt the model to other countries. Moreover, for the “guideline” strategy, we used the Dutch guideline for the treatment of degenerative meniscus tears, but international guidelines can differ. Yet, these would not alter our conclusions since the potential savings when reducing APM are substantial.

### Implications

Considerable cost savings can be achieved when the use of APM is reduced in patients with a degenerative meniscus tear, especially when APM is not performed at all. Given these findings, we recommend at least adhering to the current guidelines to avoid unnecessary costs that might be better spent on proven cost-effective treatments. However, adherence to the current guidelines is still suboptimal for the treatment of degenerative meniscus tears (9;10;29). Therefore, improving the adherence to guidelines and furthering the reduction of APM from the treatment of degenerative meniscus tears could result in substantial cost savings.

Arthroscopic knee surgery in patients over 50 years has already been deemed low-value care in multiple countries (30;31) and our findings confirm that reducing this specific low-value care can result in significant savings. The reduction in the use of APM in patients with a degenerative meniscus tear can also be viewed in a broader movement to reduce low-value treatments to combat the rising healthcare costs worldwide (32). These rising healthcare costs emphasize the importance of limiting low-value care. Reducing low-value care can be challenging, but some efforts have shown promise (33). For example, in the Netherlands, thirteen hospitals participated in an effort to reduce arthroscopic treatments in orthopedic patients over 50 years old which resulted in a reduction in arthroscopies from 9 percent to 4 percent (13). This study showed that the main barrier for orthopedic surgeons to stop performing APM is their belief in the value of the procedure and trust in their own experience. While for patients, the preference of their physician and positive experiences in their environment often result in them preferring APM over physical therapy(13). In addition, an effective method to reduce the use of a low-value treatment, like APM for patients with a degenerative meniscus tear, is usually to introduce an alternative treatment method. However, there are currently no alternative treatments apart from PT and injections that could relieve patients of the complaints they are experiencing (2). Apart from new treatments, there have been developments in the prevention of knee osteoarthritis, which could also reduce the number of degenerative meniscus tears (3). In the United States, a diet and exercise program for overweight patients with osteoarthritis was cost-effective and did not considerably increase healthcare costs as most costs were offset by a reduction in other treatment costs (34). Due to the association between body mass index and meniscus tears, this could also be effective in preventing degenerative meniscus tears (35).

In conclusion, reducing APM as a treatment option for patients with a degenerative meniscus tear results in cost savings ranging between EUR 54 million and EUR 129 million, in the Netherlands. As substantial costs can be saved it is recommended to reduce APM treatment for degenerative meniscus tears.

## Supplementary material

For supplementary material accompanying this paper visit https://doi.org/10.1017/S0266462322003361. click here to view supplementary material
