# Supplementary material for: The value of reducing arthroscopic partial meniscectomy in the treatment of degenerative meniscus tears: a budget impact analysis
Source: Int J Technol Assess Health Care. 2023 Jan 18;39(1):e7. doi: 10.1017/S0266462322003361 (PMC11574529; doi:10.1017/S0266462322003361)
Supplement: Supplementary file 1 [file S0266462322003361sup001.pdf]

Supplementary table 1: Deterministic results of yearly and total treatment cost in million Euros per strategy.

Costs	Current guideline (EUR)	APM at any time (EUR)	Non-surgical (EUR)
**Year 1**	47.84	63.83	42.93
**Year 2**	95.88	111.19	87.08
**Year 3**	139.92	154.02	128.46
**Year 4**	183.21	197.80	168.09
**Year 5**	223.88	238.85	208.33
**Total costs**	**690.74**	**765.69**	**634.89**

APM = arthroscopic partial meniscectomy

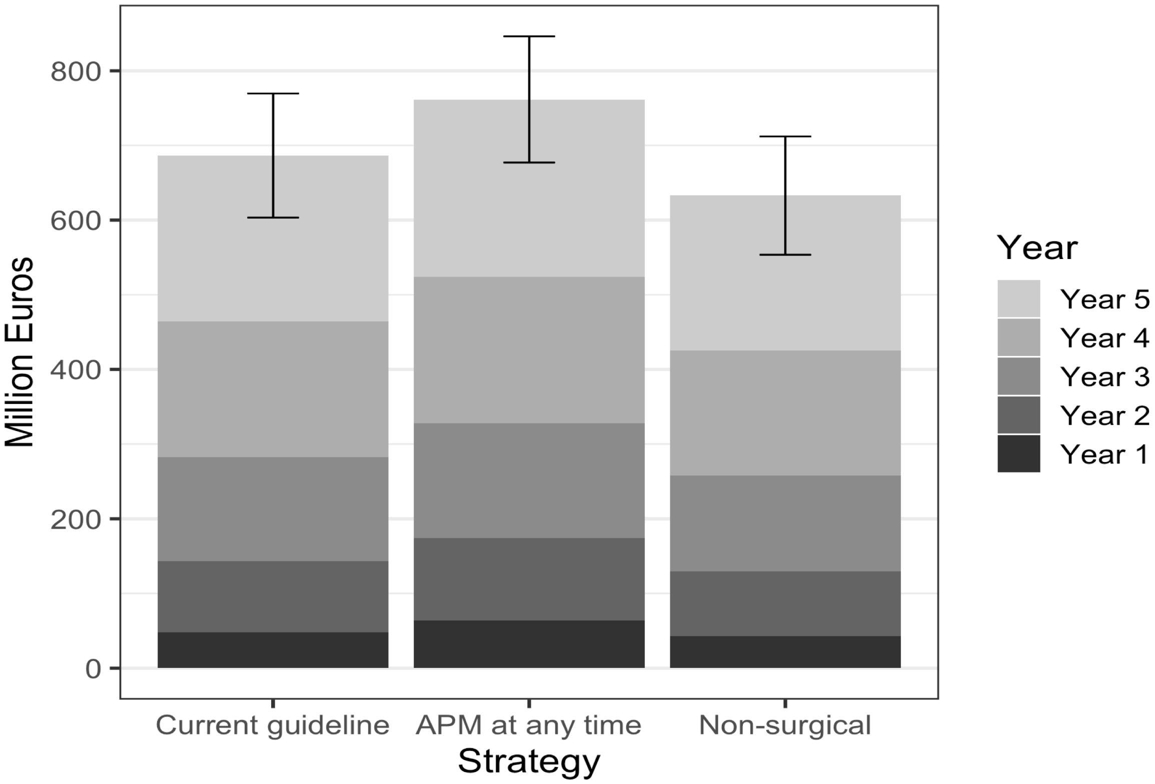

Supplementary figure 1: Yearly and overall cost per treatment strategy. Error bars represent the 95 percent confidence interval of the overall 5-year treatment cost of the strategy. APM = arthroscopic partial meniscectomy.

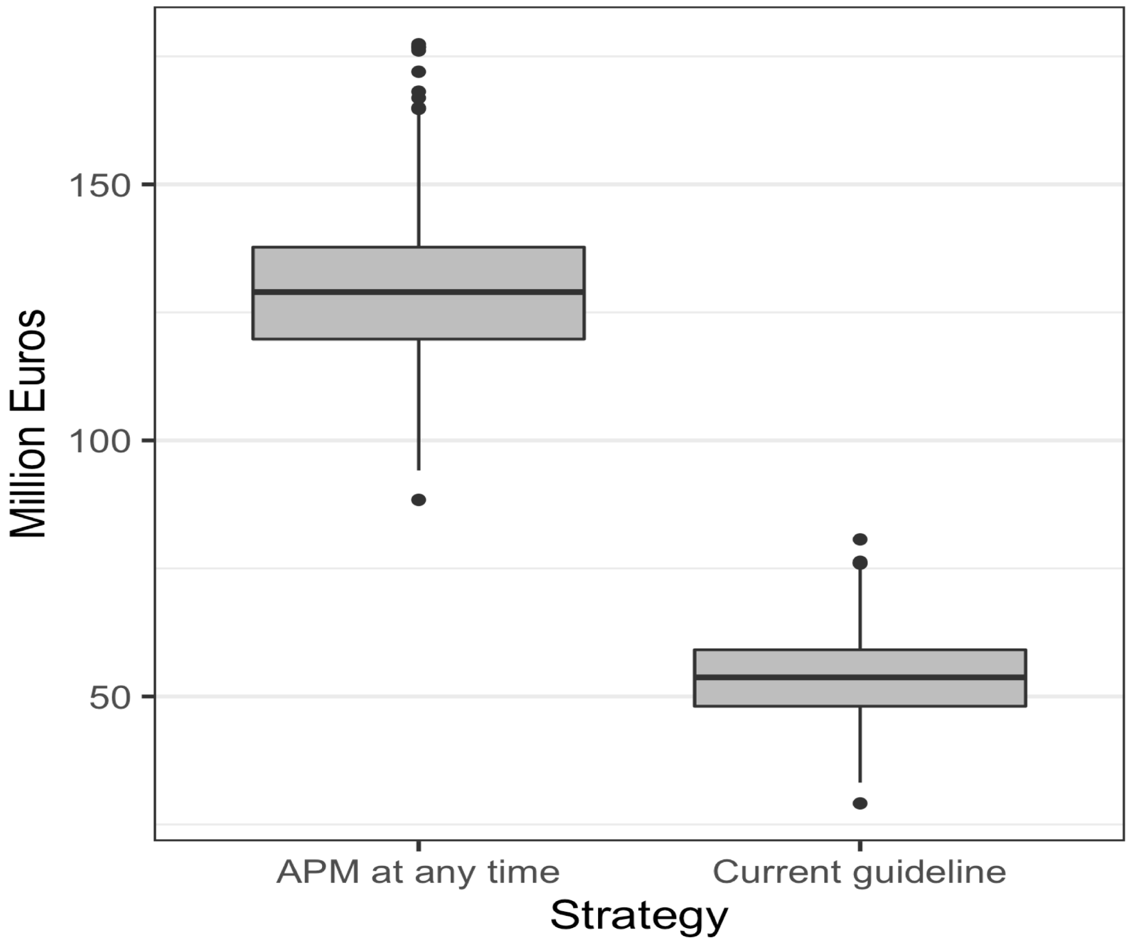

Supplementary figure 2: Boxplot of the budget impact of the 'APM at any time' and 'Current guideline' strategies compared to the 'Non-surgical' strategy over 1000 PSA iterations.
